# Environmental Factors Preceding A**β**40 Monomer to Oligomers and the Detection of Oligomers in Alzheimer's Disease Patient Serum

**DOI:** 10.1155/2012/206520

**Published:** 2012-02-08

**Authors:** Yoichi Matsunaga, Midori Suenaga

**Affiliations:** Department of Medical Pharmacology, Faculty of Pharmaceutical Sciences, Tokushima Bunri University, Yamashiro-cho, Tokushima 770-8514, Japan

## Abstract

We present here environmental factors including pH shifts, temperature, and metal ions surrounding A**β**40 monomer to precede the oligomers. We also suggest a new idea to detect A**β**40 oligomers with anti-A**β**40 monoclonal antibody using enzyme-linked immunosorbent assay. This method involves the different sensitivity of the thermal shifts between A**β**40 monomer and the oligomers. The idea is useful for the diagnostics of Alzheimer's disease to detect A**β**40 oligomers in the serum from the patients.

## 1. Introduction

Alzheimer's disease (AD) is the most common of senile dementia and characterized by memory loss, deterioration of cognitive and behavioral processes and social life, and these symptoms showed no relief through the life. The major pathological hallmark of AD is the accumulated A*β* plaques in the extracellular liquid [1] and neurofibrillary tangles in the intracellular accumulation of hyperphosphorylated and misfolded tau protein [2–4]. Among the brain lesions that are affected in AD and contain the highest number of senile plaques are the amygdala [5] and hippocampus [6]. The amygdala is involved in modulation of behavior, emotion, and memory due to its vast afferent and efferent projections. It has been reported that although lesions of amygdala alone do not appear to impair spatial learning, they potentiate hippocampus lesion-induced disruption of spatial learning [7]. The A*β* plaques are mainly composed of *β*-structured fibrils made up to *β*-amyloid protein 40/42 amino acid residues long (A*β*40/42), and they are processed from amyloid precursor protein (APP) in neurons and secreted into the interstitial fluid space (IFS) of the brain in the soluble form [8] and cerebrospinal fluid (CSF) that help to clear A*β* from IFS to the bloodstream [9, 10].

Some of misfolding mechanisms of A*β* to induce aggregation appear in AD brain. Because the aggregated A*β* is observed as extracellular structure, the concentration of A*β* in the ISF [15] and the environmental factors surrounding the protein may affect the process. Detection of the most toxic A*β* species to synapse and neuron [16] present during A*β* aggregation is a critical aspect in AD diagnostics. The molten-globule state of the protein that is a misfolding intermediate, A*β* oligomers before A*β* plaque formation is responsible for neuronal damages [17].

One possible model of direct A*β* cytotoxicity involves the cytopathic effect of amyloid fibrils, which are rich in *β*-sheets and thereby interact with cell surface receptors and result in aberrant activation of signal transduction pathways. This model in consistent with the observation of hyperphosphorylated focal adhesion kinase [18] and microtubule-associated tau protein [19]. Persistent binding and activation of cell surface receptors by amyloid fibrils may induce neuronal apoptosis. An alternative possibility of A*β*s cytotoxicity is the direct damages to the cell surface membrane to form pores and induce calcium influx by hydrolytic activity of A*β*40 oligomers [20]. Recent study reports calcium ions stimulate the formation of A*β*40 oligomers that causally implicated in neuronal toxicity of AD [21].

Many candidates of AD biomarker in blood, plasma, serum, and CSF are reported. The CSF levels of A*β*42, tau and phosphorylated tau are potential biomarkers of AD [22], and plasma measures of A*β* are of limited diagnostic value but may provide important information as a measure of treatment response [23].

Many monoclonal antibodies (moAbs) to recognize A*β*40 monomer are reported, and their epitopes are determined; however, there is no specific moAb for A*β*40 oligomers. The conformational changes of A*β*40 monomer to oligomers are induced in response to physiological environments surrounding A*β*40, and some of A*β*40 sequences are responsible to the changes. We used the moAb to react with the responsible sequence to the changes.

The A*β*40 monomer is flexible and have high free energy before exposure to environmental changes, and the moAb shows high level of signals; however, after the exposure, its flexibility is constricted and free energy became low. In the steps, some epitopes of the moAb are hidden inside the oligomers and the moAb could not bind to the epitopes, which resulted in the low level of signals. The remarkable fall down of reactivity of moAb towards A*β*40 monomer is useful to distinguish from A*β*40 oligomers, because the reactivity towards A*β*40 oligomers show no changes before and after the exposure. We applied the idea to determine A*β*40 forms in Alzheimer's disease patients serum.

## 2. Environmental Factors Surrounding A***β***40

**Figure 1:**
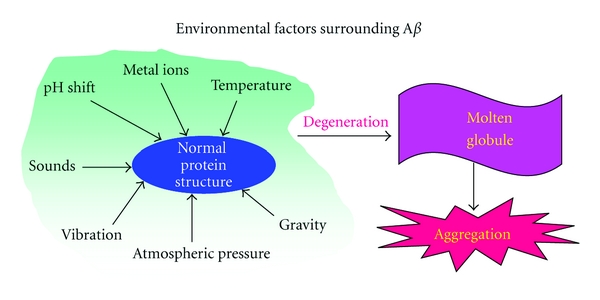
Environmental factors surrounding A*β*40 to precede A*β*40 oligomers. A*β*s are exposed to the variety of environmental factors that affect the conformational changes. These factors involve pH shift, temperature, metal ions, sounds, gravity, atmospheric pressure, and vibration. These factors might be responsible to induce soluble A*β* monomer into oligomers which is a component of neurodegenerative process.

A*β*s on the neuronal cell are exposed to various intracellular, and extracellular environmental factors in brain. A*β*s are exposed to acidic pH in lysosome and extracellular factors surrounding A*β*s involve temperature, metal ions, vibration, sounds, gravity, pressure, free radicals, lipid, concentration, and chemical chaperones [24]. These factors might be responsible to induce soluble A*β* monomers to molten-globule states of oligomers which are a component of neurodegenerative process and toxic to neuronal cells (Figure 1).

### 2.1. pH Shifts

The native conformation of A*β* in AD and PrP in Scrapie is modulated by pH shift surrounding the protein, resulting in a “molten globule” state that is less ordered than native protein and is a folding intermediate to precede amyloid protein, however, still preserve the mean overall feature of the native protein [25].

**Figure 2:**
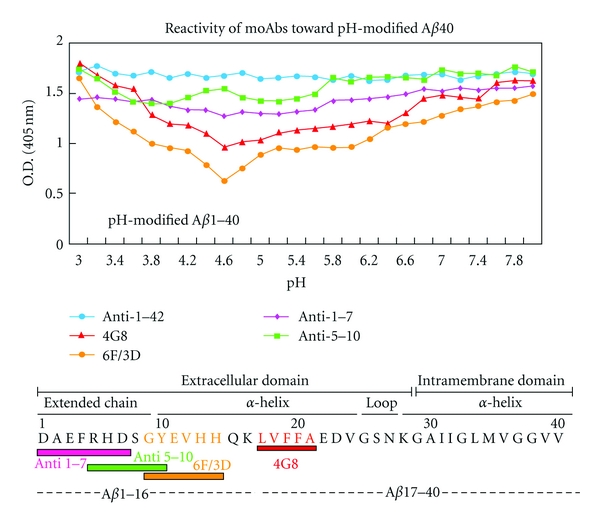
Reactivity patterns of antibodies differ after pH-modification of the A*β*40 peptide [11]. The pH-modified A*β*40 peptides (4 *μ*g/mL) at various pH values were tested for monoclonal antibody (e.g., anti-1–7 (fuchsia diamond), anti-5–10 (green square), 6F/3D (yellow circle), and 4G8 (red triangle)) and anti-1–42 (blue circle) which is a polyclonal antibody, binding at a concentration of 1 *μ*g/mL on the standard ELISA system. Absorbance was measured at 405 nm. Results are expressed as means ± SEM (*n* = 6). The anti-1–7 and anti-5–10 antibodies showed very similar reactivity towards A*β*40 incubated at various pH values, the reactivity remaining constant over the entire pH range. In constant, the 6F/3D antibody showed decreasing reactivity as the pH was lowered from pH 8.0 to 4.6, followed by a dramatic increase as the pH was lowered from pH 4.6 to 3.0. Interestingly, reactivity of the 4G8 antibody showed a profile similar to that shown by antibody 6F/3D, with the lowest signal at pH 4.6.

 In A*β*40, a critical pH to induce the conformational transition is at around pH 5, which is a mimicking of a lysozomal pH [11]. The responsible sequences to the pH shift was A*β* 9-14:GYEVHH and 17–21:LVFFA, and the Glu at position 11 is most responsible to the acidic pH shift and induce soluble A*β*40 to insoluble form [26] (Figure 2).

Scrapie is a disease of protein misfolding of cellular prion protein (PrP^c^), in which the largely *α*-helical and PrP^c^ is converted to pathological isoform of scrapie PrP(PrP^sc^), that is rich in *β*-sheet [27, 28]. PrP^sc^ is formed in caveolae and subsequently accumulated in endosomes and secondary lysosomes where the pH falls down to around pH 4-5 [29]. A proteolytically truncated form of PrP^cs^ corresponding to PrP(90–231) retains infectivity and form amyloid fibrils [30], and truncated PrP^c^(PrP90–231) with the same amino acid sequence can be converted to PrP^sc^ in both cultured cells and transgenic mouse [31].

**Figure 3:**
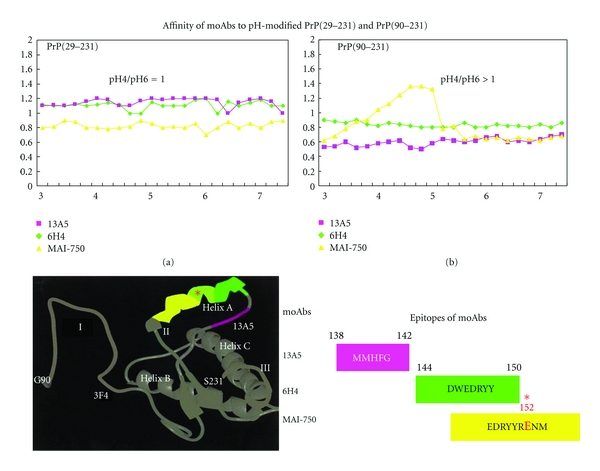
Affinity of moAbs to pH-modified PrP(23–231) and PrP(90–231) [12]. The pH dependence of the affinity of rFab 13A5 (fuchsia square), mAb 6H4 (green diamond), and mAb MAI–750 (yellow triangle) toward (a) SHaPrP(29–231) and (b) SHaPrP(90–231) after incubation at various pH values within the range 3.0–7.2 was tested. Four acidic residues are clustered in the central region within the nine residues 144–152, and rFab 13A5, mAb 6H4, and mAb MAI–750 could bind in this region. None of these antibodies showed any pH dependence of reactivity toward PrP(29–231), whereas MAI-750 showed a pH dependence around at pH 4 to 5 in its reactivity toward PrP(90–231). A further reduction in the reactivity of MAI-750 at very low pH suggests that the antibody is unable to bind to its epitope when the acidic groups are strongly protonated.

The pH dependence of PrP(90–231) was tested, and the moAbs which recognize amino acids sequence of 94–105(GTHNQWNKPSKP), 107–112(TNMKHM), and 138–142(MMHFG) of PrP^c^ showed pH-dependent reactivity. They showed high signals towards PrP(90–231) modified below pH 4.7, and the signals fall down remarkably towards PrP(90–231) modified over pH 5.2, suggesting that these regions of PrP(90–231) were sensitive to pH shifts; however, the signals from the same region of the full length of PrP(29–231) showed no sensitivity to the entire pH shifts. This pH dependence of PrP(90–231) suggests the titration of an acidic region that might inhibit the N-terminal epitopes. A similar pH dependence for a monoclonal antibody reactive to the central region of 146–154(EDRYYRENM) identified an acidic region incorporating Glu152 as a significant participant (Figure 3) [12].

### 2.2. Temperature within Physiological Limits

Temperature-induced transition of A*β*40 plays an important role in the structural transformation from *α*-helix and random coil to *β*-sheet form in aqueous solution by heating [32]. High temperature induces structural changes in A*β* (tangle and plaques) or changes in brain similar to those observed in AD [33]. The conformation of A*β*40 at 0–20°C was *α*-helical, whereas conformational changes of A*β*40 towards *β*-sheet conformation were observed at between 35–45, 60–65, and 80–85°C [13].

**Figure 4:**
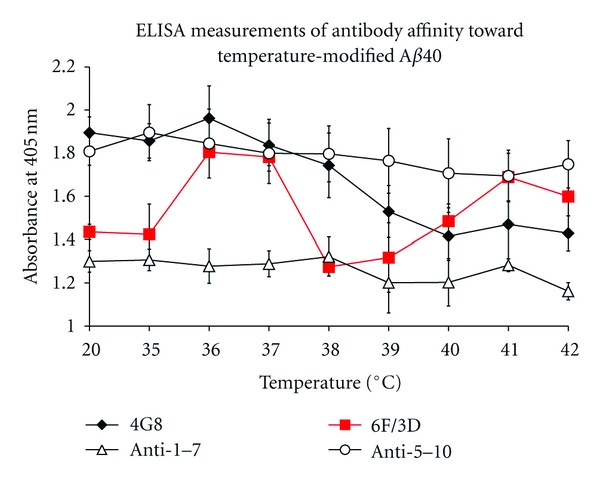
Affinity of moAbs to thermal-modified A*β*40 [13]. Enzyme-linked immunosorbent assay (ELISA) measurements of antibody affinity towards temperature-modified A*β*40 were performed. The reactivity of four antibodies as mentioned in Figure 2 (4G8, 6F/3D, anti-1–7, anti-5–10) to samples of A*β*40 that had been exposed to temperatures over the range 36–42°C at 1°C interval and at 20°C as a control, was monitored by ELISA. Statistical comparisons were performed with unpaired *t*-tests. The values are means ± SEM. Statistically significant differences versus 6F/3D were determined at each temperature (*n* = 6). For both anti-5–10 and 1–7 antibodies, the reactivity was constant through the whole temperature range from 35°C to 42°C, and no temperature-dependent difference was detected. The monoclonal antibody 6F/3D showed temperature-dependent reactivity, and the reactivity was bimodal, when the modified temperature was increased from 36 to 38°C and from 38 to 41°C. On the other hand, 4G8 showed temperature-dependent reactivity, when the temperature increased from 36 to 40°C. Thus, the 9–14 and 17–21 amino acid residues within A*β*40 peptide were sensitive to temperature changes.

The occurrence of changes within specific temperature ranges may indicate thermal specificity or adoption by A*β*40 of various conformations at wide range of heating, due to increasing intermolecular *β*-sheet structures [34]. The temperature within the physiological limits also induced the changes to A*β*40, the apparent changes were observed at 36–38°C in the amino acid residues 9–14, and the changes in the amino acid residues 17–21 were observed at 36–40°C [13], and both sequences have been reported to be involved in pH-induced conformational transition of A*β*40 [11] (Figure 4). The observation within physiological limits may occur *in vivo* with high fever over 38°C in response to inflammatory disease, and thermal stress may affect A*β*40 in our brain.

Temperature-dependent secondary structure of A*β*40, A*β*42 and A*β*28 in the solid state was also studied by simultaneous Fourier transform infrared/differential scanning calorimetry (FT-IR/DSC) microspectroscopic system. Basically, A*β*28 is composed from major *β*-sheet and minor *α*-helix with little random coil, and A*β*40 consisted of major *β*-sheet, minor random coil, and little *α*-helix, but A*β*42 mainly consisted of the predominant *β*-sheet structure. Thus, the intact A*β*s show a different secondary structures, and thermal treatment induces a similar *β*-sheet structure for A*β*40 at 45°C and for A*β*28 at 40°C; however, there was no transitional temperature for A*β*42 [35]. Actually, temperature at 45°C induced changes for A*β*40 by reducing the compositions from 37 to 20–24% for *α*-helical and random coil structures but increasing the components from 27 to 45% for intermolecular *β*-sheet structures [34]. The thermal-induced denaturation is an important factor in the structural transformation from *α*-helix/random coil to *β*-sheet in A*β*s.

### 2.3. Metal Ions

The metal ions such as Zn^2+^, Cu^2+^ were implicated in AD progression [36–38], and their interaction with A*β* in stimulating A*β* aggregation has been studied *in vitro*. The binding of metal ions induces A*β* conformation, initially rich in random coil structure to a *β*-sheet structure, favorable the partially folding intermediates. Under acidic pH, Cu^2+^, and Fe^3+^ induce drastic aggregation; however, under neutral or alkaline pH, they showed limited propensity to A*β* aggregation [39–41]. Oligomerization of the A*β* peptides can be rapidly induced in the presence of Zn^2+^ ions under physiological conditions [42–44], and, under both at alkaline and acidic pH, it could induce A*β* aggregation and form protease resistant aggregates [45]. It was suggested that A*β* possesses preferential Zn^2+^-binding sites in its N-terminal 1–16 and the metal ion interacts with His-6, -13, and -14 both at acidic and alkaline pH [46–48]. Recent reports suggests that soluble A*β* oligomers rather than matured A*β* fibrills exhibit the major neurotoxicity and these histidine residues may be a target to decrease the cytotoxicity [49] and suggested that a Pt compounds and Ru^2+^ complex may react with A*β*28 [50].

Recent report suggest the mechanisms and structures of amyloid formation by Zn^2+^ binding. Though Zn^2+^ does not affect the *β*-sheet association around the C-terminal hydrophobic region, it shifts the relative aggregation major species. As a result, Zn^2+^ coordination promotes A*β*42 aggregation leading to less uniform structures and increasing Zn^2+^ concentration slows down the aggregation rate [51].

Though there are many reports to demonstrate the neurotoxic effects and their interference with a variety of cellular and metabolic process, the pathogenesis of Al^3+^ in AD is still under debate [52, 53]. A comparative study under alkaline pH showed that Zn^2+^ and Cu^2+^ ions were much less efficient than Al^3+^ ion in stimulating the spontaneous fibril formation of A*β*s. The putative Al^3+^-chelating amino acid residues are present both in the hydrophilic segment (A*β*1–16) and in the hydrophobic core (A*β*20–35), suggesting that the Al^3+^-binding is less restricted than that for Zn^2+^ and Cu^2+^, which is confined to the N-terminal sequence [54]. 

## 3. Application of Temperature-Induced A***β***40 Conformational Changes for AD Diagnostics [14]

The earlier intervention is required in AD treatments, and the determination of clinical phase of AD in early stage is necessary. Up to date, there are no reports about useful markers of AD staging.

We exposed A*β*40 peptide to the thermal shifts and observed the reactivity pattern to moAbs with an enzyme-linked immunosorbent assay (ELISA) before and after the exposures. The signals from A*β*40 before thermal exposure fall down after the exposure, in which A*β*40 oligomerization is induced by thermal shifts.

**Figure 5:**
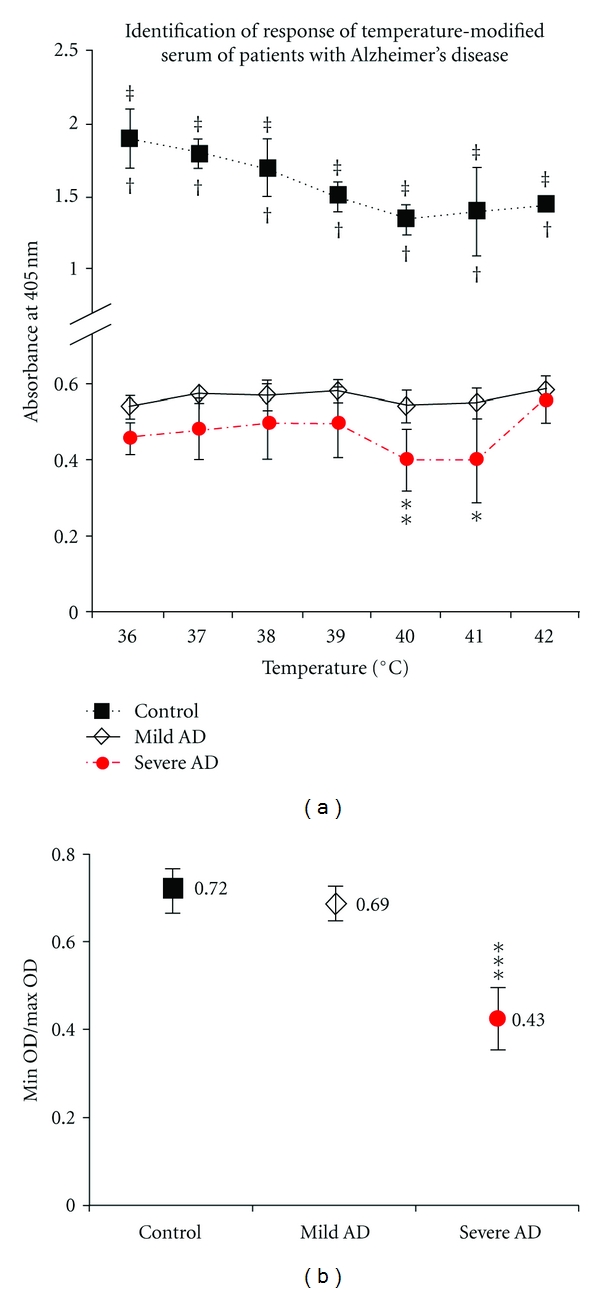
Identification of response of temperature-modified serum of patients with Alzheimer's disease [14]. According to the criteria of National Institute of Neurological and Communicative Disorders and Stroke/Alzheimer's Disease and Related Disorders, AD patients were enrolled, and the patients were classified into two groups of mild AD and severe AD with age match. MMSE score 1–9 was as severe AD and the score of 10–22 was as mild AD. Sera from two AD groups were tested for their reactivity against 4G8 with ELISA. (a) The absorbance in the severe AD was lower than that of the mild AD though the temperatures and the signals from mild AD were almost constant; however, the significant lower signal was observed in severe AD at 40°C (*P* < 0.02) and 41°C (*P* < 0.05). The signals from A*β*40 peptide as a control showed decrease as the temperature increase in a temperature-dependent manner. (b) The minimum/maximum optical density ratio of each patient's serum was calculated and obtained the average of ratios for severe and mild AD. The ratio value for patients with severe AD (0.43 ± 0.05) was significantly (*P* < 0.001) lower than that for patients with mild AD (0.69 ± 0.01). The average min/max optical density value for the synthetic A*β*1–40 peptide, used as a control, was 0.72 ± 0.02.

The clinical phase of AD was determined with MMSE, and mild AD presents MMSE over 24 and severe AD presents MMSE below 9. We used the sera from patients diagnosed with mild AD and severe AD to detect the different reactivity pattern to specific antibodies targeting A*β*17–21(4G8 moAb). The reactivity patterns of sera to 4G8 at 36–42°C was determined by ELISA. The A*β*40 peptide as a control showed the reactivity pattern in a temperature-dependent manner, and the reactivity of sera from patients with severe AD was less than that of sera from patients with mild AD though the temperatures 36–41°C and the remarkable fall down at 41-42°C were shown in severe AD, however, with no difference at 42°C (Figure 5(a)). The severity of AD is associated with greater A*β*40 aggregation. We propose that the ratio of differences of signals with ELISA between 38°C and 40°C is useful to determine the severity of AD (Figure 5(b)). The present results may be of value in staging and following up of patients with AD. 

## 4. Conclusion

Acidic environment at around pH 5, a temperature between 38-39°C within physiological limits, and Cu^2+^ and Zn^2+^ ion at neutral pH could precede A*β*40 from monomer to oligomers. The sequence of QKLVFFA is responsible of the changes, and it is crucial of A*β* oligomerization, and the sequence may be useful as a biomarker of A*β*40 oligomers in AD serum. The differences of A*β*40 conformation in AD patients serum were demonstrated as the different sensitivity of A*β*40 in response to the thermal shift, and it was detected with the moAb which recognizes QKLVFFA, corresponding to amino acids 15–21 of A*β*40/42 by ELISA. We suggest here a new diagnostic approach for AD staging by monitoring the reactivity mode of the moAb to A*β*40 before and after exposure to the thermal shift.
